# Engineered proxies and the illusion of de-extinction

**DOI:** 10.1016/j.stemcr.2025.102505

**Published:** 2025-05-15

**Authors:** Dusko Ilic

**Affiliations:** 1 Department of Women and Children’s Health, School of Life Course and Population Sciences, Faculty of Life Sciences and Medicine, King’s College London, London, UK; 2 Assisted Conception Unit, 11th Floor Tower Wing, Guy’s Hospital, Great Maze Pond, London SE1 9RT, UK

## Abstract

The recent creation of dire wolf-like canids by Colossal Biosciences marks a technical achievement in genome editing and synthetic embryology. But the project also demands a reevaluation of what we mean by “de-extinction”—and whether a phenotypic approximation constitutes species restoration.

Colossal Biosciences (TX, USA; https://colossal.com/) announcement that it has successfully engineered and birthed three pups with phenotypic features reminiscent of the extinct dire wolf (*Aenocyon dirus*) has garnered significant attention. Using comparative genomics and multiplex CRISPR editing, researchers modified a gray wolf (*Canis lupus*) cell line to express twenty genetic edits across fourteen loci, informed by ∼91% of the reconstructed dire wolf genome. The resulting animals exhibit increased body mass, cranial robustness, and light coat pigmentation—traits consistent with the extinct species. But despite headlines declaring the dire wolf’s return, what has been achieved is not resurrection, but simulation: a synthetic proxy designed to mimic phenotype, not to replicate genotype.

This distinction has profound implications for both the scientific and ethical framing of de-extinction. Genotype—the full genomic blueprint—is not simply a background variable. It encodes the developmental, behavioral, immunological, and ecological identity of a species. Phenotype, shaped by both genotype and environment, is its expression. In Colossal’s dire wolves, selected traits were prioritized for viability and recognizability, while potentially deleterious variants (e.g., pigmentation-associated mutations linked to sensory deficits) were excluded. The outcome is an engineered animal that resembles the dire wolf in form, but not in totality.

Such work highlights the practical shift in de-extinction strategy from full genomic synthesis—still far beyond reach for mammalian genomes—to targeted phenotypic reconstruction. Even George Church, Colossal co-founder and pioneer in synthetic genomics, acknowledges that whole-genome synthesis at the scale of the ∼3 Gbp mammoth genome remains a “cold-fusion-level” challenge. Instead, partial phenotypic fidelity—what Colossal’s team terms “functional de-extinction”—has become the operational endpoint: a pragmatic compromise that prioritizes outward resemblance and selected functional traits over complete genomic or ecological resoration.

## Technical concerns

**Figure 1:**
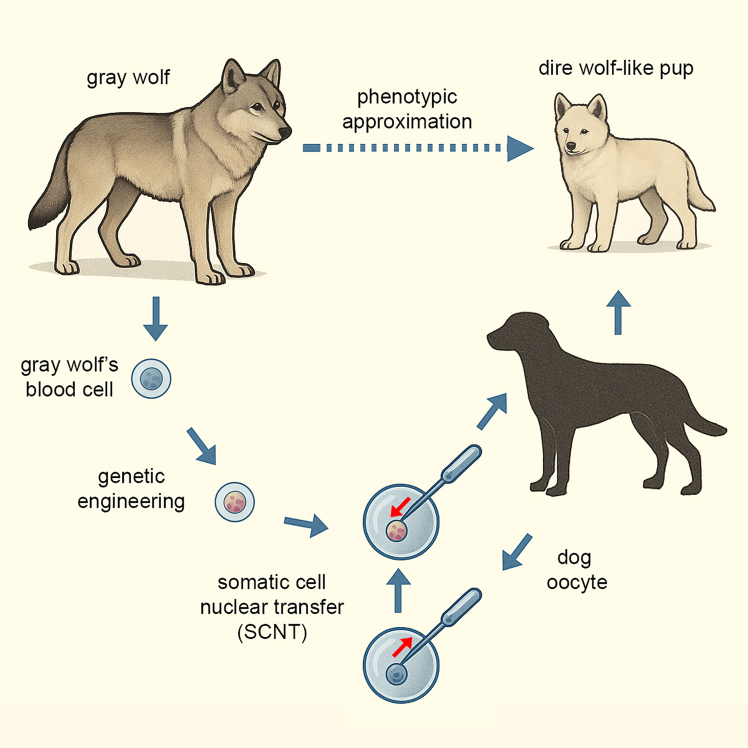
Cloning strategy used to generate dire wolf-like canids Somatic cells from a gray wolf were genetically engineered at approximately twenty loci using multiplex CRISPR editing to introduce traits inferred from the extinct dire wolf genome. The edited nuclei were transferred into enucleated oocytes from domestic dogs via somatic cell nuclear transfer (SCNT). The resulting embryos were implanted into surrogate dog mothers and developed into live pups exhibiting selected phenotypic traits (e.g., cranial robustness, body mass, and pigmentation) consistent with dire wolf morphology. The outcome represents phenotypic approximation rather than genomic or ecological resurrection.

Colossal’s project does not represent the culmination of stem cell-based developmental and/or conservation biology (Stanton et al., 2018), as originally claimed, but rather reflects a more traditional cloning approach with synthetic augmentation. While the company has not released peer-reviewed data, publicly available sources indicate that somatic blood cells were genetically edited at approximately twenty loci and then used in somatic cell nuclear transfer (SCNT) (Max, 2025). These nuclei were inserted into enucleated dog oocytes, and the resulting embryos were gestated in large domestic dogs, not wolves (Figure 1). The yield was low: 2 live births from 45 embryos, reflecting persistent challenges in *ex vivo* developmental biology and post-edit viability. This method, reminiscent of the technique used in Dolly the sheep, bypasses pluripotency induction or *in vitro* gametogenesis entirely.

This distinction is significant. The omission of induced pluripotent stem cell derivation or artificial gamete generation narrows the scope of technological novelty while raising well-documented concerns about the health and viability of cloned animals. SCNT-derived mammals frequently suffer from epigenetic abnormalities, metabolic disorders, and shortened lifespans. The health of Colossal’s dire wolf-like offspring has not been independently assessed or disclosed in detail. Without such data—ideally in a peer-reviewed format—it is impossible to determine whether the engineered animals are viable, fertile, or behaviorally analogous to their extinct analogs.

The technical, ethical, and biological complexities of cloning as a de-extinction tool are not merely theoretical. A striking case is that of the bucardo (*Capra pyrenaica pyrenaica*), a subspecies of Spanish ibex that was declared extinct in 2000. After the death of the last known individual, scientists attempted to clone the animal using nuclei from preserved tissue and goat oocytes. In 2003, 57 embryos were implanted. Only one resulted in a live birth—and the cloned bucardo survived for less than 10 min due to a fatal lung defect. This event marked the first and only time a species has been brought back from extinction, only to go extinct again. The bucardo’s brief return highlights the biological limitations, raising unresolved ethical questions about animal welfare, viability, and purpose of “de-extinction.”

Despite its symbolic appeal, SCNT remains a technically fragile and inefficient method for species restoration. The bucardo’s brief reappearance is not an exception but a representative case. Across mammalian species, SCNT consistently exhibits low efficiency, with most attempts resulting in embryonic failure, pregnancy loss, or postnatal abnormalities.

This lack of transparency underlines a critical need: any claim of functional de-extinction must be accompanied by rigorous, open peer evaluation of the methods used, the phenotypic expression achieved, and the physiological integrity of the resulting animals. Until then, the “dire wolves” remain closer to concept demonstrations than to viable ecological actors.

## Conceptual concerns

The broader concern is conceptual. Public and media discourse around “bringing back” extinct species obscure the reality that no complete dire wolf genome has been synthesized, no native behaviors reconstituted, and no ecological reintegration achieved. The engineered pups have never interacted with conspecifics, learned species-typical behaviors, or occupied their ancestral ecological niche. They are constructed organisms, not revived ones.

This raises essential questions for the field of synthetic biology. At what point does a heavily edited organism with partial trait recovery become a new species? How do we distinguish between de-extinction and *de novo* organism design? And what frameworks should be used to evaluate success—not only technical, but ecological and ethical?

The dire wolf project also mirrors a growing commercial trend in synthetic biology: dual-use platforms that link scientific ambitions with monetizable applications. Colossal has positioned its work as ecologically restorative, yet its funding model, media strategy, and investor outreach (including celebrity partnerships and cinematic branding) signal an additional aim: to transform charismatic megafauna into biotech showcases and potential intelectual property assets. This model echoes fictional scenarios from speculative literature—most notably Margaret Atwood’s Oryx and Crake—where hybrid animals are engineered for commercial and symbolic value rather than ecological function (Atwood, 2003).

As scientists, we must ensure that the rhetoric surrounding engineered proxies does not exceed what the underlying biology supports. “De-extinction” should not become a synonym for “phenotypic mimicry.” Instead, a more accurate vocabulary—such as “synthetic proxies” or “engineered simulacra”—would better capture what has been achieved, while leaving room for rigorous distinctions as technologies evolve.

## Conclusion

Colossal’s work advances genome editing and mammalian embryology in significant ways, and its technical achievements deserve recognition. But science progresses through clarity, not spectacle. As we build the tools to shape life with increasing precision, we must also build the conceptual frameworks to define what, exactly, we are creating.

## Declaration of interests

The author declares no competing interests.
